# Interactions between Apolipoprotein E Metabolism and Retinal Inflammation in Age-Related Macular Degeneration

**DOI:** 10.3390/life11070635

**Published:** 2021-06-29

**Authors:** Monica L. Hu, Joel Quinn, Kanmin Xue

**Affiliations:** 1 Centre for Eye Research Australia, Royal Victorian Eye and Ear Hospital, East Melbourne, VIC 3002, Australia; monica.hu.011@gmail.com; 2 Nuffield Laboratory of Ophthalmology, Nuffield Department of Clinical Neurosciences, University of Oxford, Oxford OX3 9DU, UK; joel.quinn@ndcn.ox.ac.uk; 3 Oxford Eye Hospital, Oxford University Hospitals NHS Foundation Trust, Oxford OX3 9DU, UK

**Keywords:** age-related macular degeneration, apolipoprotein E, amyloid-beta, retinal inflammation, drusen

## Abstract

Age-related macular degeneration (AMD) is a multifactorial retinal disorder that is a major global cause of severe visual impairment. The development of an effective therapy to treat geographic atrophy, the predominant form of AMD, remains elusive due to the incomplete understanding of its pathogenesis. Central to AMD diagnosis and pathology are the hallmark lipid and proteinaceous deposits, drusen and reticular pseudodrusen, that accumulate in the subretinal pigment epithelium and subretinal spaces, respectively. Age-related changes and environmental stressors, such as smoking and a high-fat diet, are believed to interact with the many genetic risk variants that have been identified in several major biochemical pathways, including lipoprotein metabolism and the complement system. The *APOE* gene, encoding apolipoprotein E (APOE), is a major genetic risk factor for AMD, with the *APOE2* allele conferring increased risk and *APOE4* conferring reduced risk, in comparison to the wildtype *APOE3*. Paradoxically, *APOE4* is the main genetic risk factor in Alzheimer’s disease, a disease with features of neuroinflammation and amyloid-beta deposition in common with AMD. The potential interactions of APOE with the complement system and amyloid-beta are discussed here to shed light on their roles in AMD pathogenesis, including in drusen biogenesis, immune cell activation and recruitment, and retinal inflammation.

## 1. Introduction

Age-related macular degeneration (AMD) is one of the leading causes of severe visual impairment worldwide [1], affecting 8.7% of the population, with a global burden projected to reach 288 million people in 2040 [2]. It irreversibly affects the macular region of the retina, the area responsible for central vision, impacting on quality of life, particularly in the elderly population. Early-stage AMD is characterised by the accumulation of drusen (extracellular, yellow-white lipid and proteinaceous deposits under the retina), while late-stage AMD can be classified as ‘dry’ or ‘wet’ [3]. Geographic atrophy (GA) (dry or non-exudative AMD) features progressive loss of retinal pigment epithelium (RPE), photoreceptors and choriocapillaris. About 10–15% of AMD patients develop neovascular (wet or exudative) AMD, which is characterised by choroidal neovascularisation (CNV) driven by vascular endothelial growth factor (VEGF) production, and leads to sequelae such as subretinal haemorrhage, exudation and fibrosis. While sight loss from neovascular AMD can be reduced by intravitreal injections of anti-VEGF agents, there is currently no treatment for GA, the predominant form of the disease. Antioxidant supplementation (with vitamins E and C, lutein, zeaxanthin, zinc and copper) have been shown to reduce the risk of progressing to advanced AMD in patients with early to intermediate AMD [4,5].

While the prevalence of AMD increases considerably with age, there is a clinical distinction between the appearance of ‘normal’ aged retina and pathological aging associated with more rapid progression to advanced atrophic or neovascular AMD. The severity of AMD is clinically graded based on the size of drusen and the presence of pigment abnormalities found within 2 disc diameters of the fovea in either eye [6]. The presence of only drupelets (small drusen ≤ 63 μm) and having no pigment abnormalities is considered normal aging [6]. Early AMD is defined by the presence of medium drusen (63–125 μm) and no pigment abnormalities, while intermediate AMD is defined by the presence of large drusen (>125 μm) and/or pigment changes such as hypo- or hyperpigmentation [6]. Advanced AMD is defined by the presence of neovascular AMD or geographic atrophy. This classification scheme was formulated based on the finding that the presence of medium and large drusen predicts a significant risk of progression to advanced AMD [6], highlighting the key, albeit unclear, role that lipoproteinaceous deposits play in AMD pathogenesis.

### 1.1. Types of Drusen

**Figure 1:**
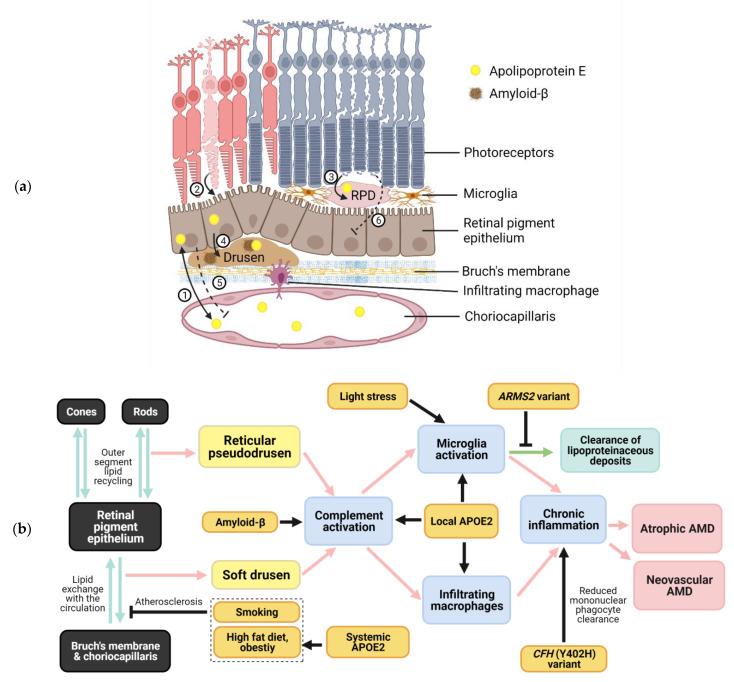
Schematics of age-related macular degeneration (AMD) pathomechanism. (**a**) Deposition of drusen (also known as basal linear deposits or soft drusen) and reticular pseudodrusen (RPD) in the retina. Drusen and RPD form as a result of abnormal lipid cycling between the circulation and retinal pigment epithelium (RPE) (1) and between the photoreceptor outer segments and the RPE (2 and 3), respectively. APOE and amyloid-β derived from the RPE locally, or from systemic circulation, contribute to the composition of drusenoid deposits (4). Drusen prevents normal lipid exchange between the RPE and choriocapillaris across Bruch’s membrane (5), while RPD blocks the normal endocytosis of rod outer segment by the RPE (6), leading to ‘vicious cycles’ of drusenoid material build up. These lipoproteinaceous deposits activate the complement system, promoting the recruitment of macrophages and microglial activation. (**b**) A model for AMD pathogenesis integrating key risk factors. Unbalanced lipoprotein exchange results in the formation of drusenoid deposits, which in turn act as foci for retinal inflammation through interactions between APOE2, amyloid-β and the complement cascade. Microglial activation and infiltrating macrophage activity promote a milieu of chronic inflammation under the influence of genetic polymorphisms. This leads to RPE and photoreceptor degeneration, and eventual advanced AMD features (geographic atrophy and, in some cases, choroidal neovascularisation). Abbreviations: APOE2, apolipoprotein E2; *CFH*, complement factor H; *ARMS2*, age-related macular degeneration susceptibility gene 2. Created with BioRender.com.

Basal linear deposits (BLinD) or soft drusen (when BLinD become confluent) are one of the hallmarks of AMD [7,8]. BLinD are deposited between the basement membrane of the RPE and the inner collagenous layer of Bruch’s membrane (BrM) [9] (Figure 1a). They are lipid-rich, with about 40% of druse volume constituting lipids—particularly esterified cholesterol and phosphatidylcholine [10] (Table 1). Over 129 different proteins have been identified in drusen [11], including apolipoproteins (E, B, A and C), amyloid-beta (Aβ), complement factors (C3, C5, C8, C9), complement factor H and vitronectin [10,12]. However, as drusen can also develop as part of normal aging, clinical criteria have been developed to define pathological drusen based on their size, location and morphology [6].

Distinct from sub-RPE soft drusen/BLinD are reticular pseudodrusen (RPD), also known as subretinal drusenoid deposits (SDD). They are so-named due to their location in the subretinal space between the photoreceptors and RPE. Appearing as net-like yellow-white lesions best appreciated by near-infrared reflectance or blue light autofluorescence imaging, RPD are associated with reduced retinal sensitivity [13,14]. The presence of RPD in AMD represents a major risk factor for progression to geographic atrophy (with odds ratio of 2.42; 95% CI, 1.80–3.24; *p* < 0.001) and, to a lesser extent, exudative AMD (odds ratio 1.21; 95% CI, 0.87–1.7; *p* = 0.26) [15,16]. While the compositions of RPD and BLinD have many similarities, RPD are notably different by containing relatively high levels of unesterified cholesterol, APOE and opsins which are likely derived from rod photoreceptor outer segment shedding [8,13,17,18] (Table 1).

### 1.2. Risk Factors for AMD

A wide variety of factors contribute to AMD risk, with age being the strongest risk factor. The prevalence of early AMD in people aged 55–59 years is 3.5%, increasing to 17.6% in people 85 years or older, while the prevalence of advanced AMD in each of these age groups is 0.1% and 9.8%, respectively [19]. Age-related alterations in the RPE, BrM and choriocapillaris are suggested to predispose individuals to the effects of further insults by environmental and genetic risk factors [20] (Figure 1b). Lifestyle or environmental factors, such as smoking (two-fold increased risk) and a high-fat diet, are associated with the development or progression of AMD and are thought to contribute to a pro-inflammatory environment via retinal oxidative stress [21]. Oxidative stress appears to drive the breakdown of the outer blood–retinal barrier, which is formed by tight junctions between RPE cells, leading to the infiltration of immune factors/cells and choroidal neovascularisation or atrophy [22]. Chronic oxidative stress in AMD may also cause impaired RPE autophagy, a physiological function that normally prevents the accumulation of oxidatively damaged organelles [23]. Drusen formation appears to be associated with chronic local inflammatory and immune-mediated events at the interfaces between the RPE and BrM, and between the RPE and photoreceptors, leading to outer retinal degeneration.

AMD has a strong genetic component. A US twin study estimated the heritability of AMD to be 71% in advanced AMD and 46% in overall AMD grades [24]. In a genome-wide association study, 52 common and rare variants across 34 genetic loci were found to explain 27.2% of disease variability and also explain over half of the genomic heritability of AMD [25]. Uncovering these associations with genetic variants, and subsequently exploring their roles via functional studies, have offered great insight into the complex pathobiology of AMD and its potential mechanistic pathways, including the interactions of genetic variants with environmental factors. Many of the AMD-associated alleles affect genes involved in lipid metabolism or transport (e.g., *APOE*, *APOC2*, *ABCA1*, *CETP*) and complement pathways (*CFH, C2*, *C3*, *C4A*, *C4B*, *CFB*, *CFI*). The additional risk of transformation from atrophic to exudative AMD may be linked to alleles involving extracellular matrix remodelling enzymes (e.g., *ADAMTS9*, *LOXL2*, *MMP9*).

Of particular interest to understanding AMD, and how its risk factors tie together, is the relationship between lipoprotein metabolism and inflammation in the retina. *APOE* is a significant genetic risk factor for AMD and encodes a lipid transport protein found in drusen that has been implicated in several AMD pathogenic pathways, including interactions with the complement pathway and Aβ oligomerisation. This review will explore the evidence for mechanistic links between APOE isoforms, Aβ deposition and complement activation in the retina in order to shed light on the pathogenesis of AMD.

## 2. Apolipoprotein E

Apolipoprotein E (human APOE; murine ApoE) has not only been implicated in the pathogenesis of AMD, but also Alzheimer’s disease (AD) and atherosclerosis [26,27,28]. Two single nucleotide polymorphisms (SNPs) in the *APOE* gene lead to amino acid substitutions at positions 112 and 158 in the APOE protein. Combinations of these two SNPs give rise to three allelic variants: *APOE3* (Cys112 Arg158), the most common (wildtype) allele with a frequency of ~80%; *APOE4* (Arg112 Arg158) with a frequency of 15%; and *APOE2* (Cys112 Cys158) with a frequency of 8% [28,29]. Analyses across different populations have demonstrated that *APOE2* is associated with increased risk for AMD (odds ratio 1.124–1.83), while *APOE4* is associated with reduced risk (odds ratio 0.43–0.81) [26,29,30,31,32]. However, this association was not found in Chinese populations [33,34]. Paradoxically, in AD, *APOE4* is a major genetic risk factor, while *APOE2* is a protective allele [27].

APOE, like other apolipoproteins, functions systemically as a plasma lipid transport protein that binds cholesterol (both esterified and unesterified) and lipids. It is involved in reverse cholesterol transport, as well as clearing remnants of very low-density lipoprotein (VLDL) and chylomicrons by the liver, the tissue in which it is most highly expressed [35]. It is a ligand for the low-density lipoprotein (LDL) receptor, as well as LDL receptor-related proteins (LRP1, LRP2 and LRP8). The single amino acid substitutions that result in the E2 and E4 isoforms cause modifications to the binding properties of APOE. Due to an Arg158Cys substitution, the E2 isoform exhibits impaired receptor-binding activity (only 1%) compared with E3 and E4 [36] with consequent diminished clearance of lipoprotein remnants from plasma [37]. Homozygosity for *APOE2* thus accounts for over 90% of cases of type III hyperlipoproteinaemia, although due to the low penetrance of this disorder, less than 5% of *APOE2* homozygotes (about 1% of the population) develop it [35]. This manifests as elevated plasma cholesterol and triglyceride levels, and increased atherosclerotic risk. The Cys112Arg substitution that results in APOE4 leads to preferential binding to VLDL, as opposed to preferential binding to HDL by the E3 and E2 isoforms, and is associated with a more pro-atherogenic lipoprotein–cholesterol distribution that also increases cardiovascular disease risk [38]. The pathological effects of APOE4 in neural tissue, particularly its role as an Aβ-binding protein, have been explored extensively in the context of understanding its role in AD (see 4. Interactions between Amyloid-β and Apolipoproteins).

It is not known why *APOE4*, which acts detrimentally in the brain, seems to offer protection in the retina—and why the opposite is true for *APOE2*. In the retina, *APOE* is expressed locally by the RPE and Müller glia and is an abundant constituent of drusen in both AMD and non-AMD patients [39]. APOE is secreted from both apical and basal surfaces of RPE, and is thought to play a role in lipid trafficking and lipid efflux through BrM to the choriocapillaris [40]. APOE has been found to accumulate in the basal cytoplasm of RPE cells near drusen [39], in addition to in drusen itself. This accumulation is thought to mainly be a result of local *APOE* expression, with a possible minor contribution from extravasated particles from choroidal circulation [39]. With cholesterol being a key constituent of drusen, the function of APOE as a cholesterol and lipid transporter likely plays a key role in drusen formation.

Rodent models provide some insight into the function of APOE and its isoforms in the retina. In addition to elevated serum cholesterol levels, ApoE-deficient mice demonstrate thickened BrM with disorganised elastic lamina, retinal function abnormalities as measured by electroretinography [41], and the accumulation of membrane-bounded material ultrastructurally similar to BLinD [42]. The *APOE3*-Leiden transgenic mouse carries a dysfunctional mutation of *APOE3* that, in humans, causes autosomal dominant hyperlipoproteinaemia and early-onset atherosclerosis. On a high-fat diet, this mouse strain develops basal laminar deposits (BLamD, extracellular deposits between the RPE cell membrane and its basement membrane) but not BLinD/soft drusen [43]. Malek et al. [44] generated a murine model of AMD using *ApoE* targeted replacement (*TRE*) mice expressing the three human isoforms (*TRE2*, *TRE3*, *TRE4*). Aged *TRE* mice given a high-fat cholesterol-rich diet were found to recapitulate hallmark features of AMD such as drusenoid deposits resembling BLamD and BLinD, thickened BrM, regions of RPE atrophy and choroidal neovascularisation. In contradiction to results from human epidemiological studies, these features were the most severe in *TRE4* mice, followed by *TRE2* mice, and were minor in *TRE3* mice. Irrespective of isoform expression, advanced age or a high-fat cholesterol-rich diet alone were insufficient to induce these AMD features.

CX3CR1 is a receptor normally expressed by retinal microglia and functions to maintain microglial quiescence. Due to a lack of inhibitory Cx3cl1/Cx3cr1 (ligand/receptor) signalling, *Cx3cr1*-deficient mice demonstrate subretinal inflammation and photoreceptor degeneration without drusen formation, particularly on exposure to age, light or laser injury. This chronic subretinal inflammation develops as a result of subretinal mononuclear phagocyte (MP) accumulation, which is dependent on local ApoE overexpression by the MPs [45]. Further studies in *TRE* mice by Levy et al. [46] expanded on this model to provide evidence in support of the pathogenic role of *APOE2* and protective role of *APOE4* in subretinal inflammation. Compared with *TRE3* and *TRE4* mice, *TRE2* mice developed significantly greater age-related subretinal MP accumulation, photoreceptor loss and exaggerated choroidal neovascularisation after laser injury. The MPs of *TRE2* mice were found to express a significantly higher level of ApoE and demonstrated ApoE2-dependent immune activation and cytokine production. In contrast, the *APOE4* allele (in *Cx3cr1*-deficient mice) was found to be protective through reduced ApoE expression and subretinal MP accumulation, compared with *APOE3*. These results highlight the importance of differential behaviour of APOE isoforms in retinal inflammation, as well as potentially distinct pathogenic roles played by systemic versus local APOE in AMD pathogenesis when taken in the context of the work by Malek et al. above (Figure 1b).

## 3. Interactions between Apolipoproteins and Complement Factors

The complement system, an integral arm of innate immunity, is genetically and functionally implicated in AMD pathogenesis. Systemically, the complement cascade can be activated by any of the classical, alternative and lectin pathways, and results in the assembly of the membrane attack complex (MAC), a membrane pore that can cause cell death. Complement components can act as anaphylatoxins that promote inflammation by recruiting immune cells (C3a and C5a), or as opsonising particles to aim phagocytosis by myeloid cells (C3b and C4b). While the liver is the major synthetic source of circulating complement, the RPE is also a known source of complement factors and regulators [47]. AMD patients demonstrate elevated serum complement levels, but evidence from clinical trials suggests locally produced complement may play a stronger role in AMD pathogenesis due to the comparative success of intraocularly administered trial drugs over those systemically administered [48]. A large variety of complement factors and regulators are found in drusen and are hypothesised to contribute to chronic inflammation and immune-mediated events at the RPE/BrM interface [49]. Additionally, enhanced phagocytic activity of macrophages when engulfing dying RPE cells is associated with a change in the expression of complement genes, such as *C1QA*, *C2* and *C4A* [50]. An impaired ability of RPE cells and macrophages to engulf cellular debris is thought to contribute to the accumulation of drusen.

From this perspective, the complement system seems a promising therapeutic target, but the development of an effective complement modulator for AMD is ongoing. Despite initial phase II clinical trial results showing reduced GA progression, intravitreal delivery of the factor D inhibitor lampalizumab, which blocks activation of the alternative pathway, failed to demonstrate this outcome in phase III trials [51]. Eculizumab, a systemic C5 inhibitor, also did not significantly reduce GA progression [52]. Intravitreal pegcetacoplan, a selective C3 inhibitor, and intravitreal avacincaptad pegol [53], a C5 inhibitor, have shown potential for halting GA progression and have advanced to phase III trials (NCT03525600 and NCT04435366, respectively). Clinical trials of gene therapy targeting the complement system have also commenced, with a complement factor I gene augmentation construct (NCT04437368, NCT04566445) and an antisense oligonucleotide against complement factor B (NCT03815825) entering phase II trials.

Of AMD genetic risk variants associated with the complement system, the highest risk is associated with the *CFH*(Y402H) variant (odds ratio 2.45–5.57 [54]). *CFH* encodes complement factor H (CFH), a negative regulator of the alternative pathway. The *CFH*(Y402H) variant is a common polymorphism causing a Tyr402His substitution, which leads to reduced binding affinity to BrM, C-reactive protein and oxidised phospholipids [55,56,57]. The exact pathomechanism of this risk variant remains to be clarified, but as individuals homozygous for *CFH*(Y402H) demonstrate elevated choroidal MAC and C-reactive protein deposition [58,59], *CFH*(Y402H) is thought to have reduced immune inhibitory function. Aged transgenic *CFH*(Y402H) mice on a high-fat diet develop features similar to AMD, including vision loss, increased RPE stress, and increased BLamD [60]. They also demonstrate elevated levels of apolipoproteins B48 and A1 in the RPE/choroid. These features were not found in normal *CFH* transgenic mice, or *CFH*(Y402H) mice that were young or fed a normal diet, thus suggesting interplay between complement activation and lipid metabolism.

In a human RPE cell-culture model, exposure to serum containing C1q led to the formation of sub-RPE deposits rich in APOE and other known drusen components, such as vitronectin and amyloid P [61]. This implicated an inflammatory contribution from complement activation via the classical pathway, stemming from C1q binding to ligands in these deposits and leading to MAC formation. Furthermore, exposure of cultured RPE cells to serum depleted of C1q to activate the alternative pathway led to an increase in cell-associated APOE levels [62]. This increase was found to be dependent on MAC formation, and cell-surface APOE was found to colocalise with MAC. Drusen in human eyes also demonstrated colocalisation of APOE with MAC [62]. APOE isoform-specific differences in these interactions have not been explored, although all isoforms have demonstrated the ability to act as checkpoint inhibitors of the classical complement cascade by binding directly to C1q in vitro [63]. This could suggest the upregulation of APOE that occurs with complement challenge may be related to its function as a negative regulator. Overall, further studies are needed to examine how these interactions between APOE and the complement system lead to AMD pathogenesis.

## 4. Interactions between Amyloid-β and Apolipoproteins

Amyloid-beta (Aβ) peptides are present in drusen and its precursor, amyloid precursor protein (APP), is expressed by the RPE and retinal ganglion cells [64,65,66], but their roles in retinal degeneration remain unclear. Aβ peptides are derived from the proteolytic cleavage of APP, a transmembrane glycoprotein: APP is first cleaved by β-secretase (or BACE1) at a site adjacent to the cell membrane, yielding a soluble fragment (sAPPβ) and a membrane-bound fragment (C99); C99 is further cleaved by the γ-secretase complex within the membrane to generate monomeric Aβ [67]. Depending on the site of γ-secretase cleavage, Aβ peptides of various lengths are produced, with the most abundant forms being the Aβ_1–40_ and Aβ_1–42_ variants. Aβ_1–42_ monomers are particularly susceptible to aggregation, oligomerisation and fibril formation and are highly enriched in the senile plaques of AD patients.

In addition to its suggested contribution to AD pathogenesis, several lines of evidence indicate Aβ may be relevant to ocular aging and AMD. The majority of studies have focused on retinal immunostaining of mouse, rat and human donor eyes, with the first reports finding Aβ and APP in aged retinae [68]. In healthy mice and humans, there appears to be an age-dependent increase in Aβ deposition in the retina. Notably, the RPE/Bruch’s membrane interface, photoreceptor outer segments, and inner and outer retinal vasculature showed increased immunoreactivity to Aβ antibodies with age [69].

The first analysis of Aβ in AMD showed that Aβ colocalises with activated complement components in drusen, forming spherical substructures termed ‘amyloid vesicles’ [64]. Aβ is found predominantly at the surface of these vesicles in the form of protofibrils and mature fibrils [70,71]. Dentchev et al. showed that Aβ is present in the drusen of some AMD retinae but not in the drusen of healthy donors, with Aβ appearing most frequently in drusen at the peripheral regions of geographic atrophy [65]. However, other studies later found that Aβ oligomers are present in both AMD and non-AMD eyes with drusen, but not in healthy age-matched eyes lacking drusen [72]. These ‘amyloid oligomer cores’ were consistent in size (~15 µm) and formed a major constituent of smaller drusen, while in large drusen, several cores were detected, suggesting a fusion of smaller drusen. In addition, the cores did not colocalise with the Aβ vesicles previously described, although this may be attributed to the masking of the Aβ monomer epitope upon oligomer formation and account for the inconsistency with Dentchev et al.’s findings [72]. Further immunostaining experiments probing the different Aβ species in drusen suggest that Aβ oligomers are present in drusen-containing tissue, although not all drusen contain oligomer cores [71].

One proposed sequence of events is that Aβ oligomers form adjacent to the RPE/BrM boundary, followed by Aβ interactions with complement components and lipids, eventually forming the mature fibrils that comprise amyloid vesicle surfaces [73]. The RPE has been identified as a major source of Aβ in the retina, due to positive staining for APP in the cytoplasm of RPE, and Aβ in the RPE above drusen [64,70]. Furthermore, cultured RPE cells express both APP and Aβ, as well as the key enzymes involved in Aβ metabolism [64,74]. In addition to the role of RPE in Aβ production, several studies have also investigated the effects of Aβ peptides on RPE signalling and health. Cultured RPE cells exposed to Aβ increased expression of pro-angiogenic VEGF and decreased expression of anti-angiogenic PEDF [74]. Moreover, conditioned media from Aβ-exposed RPE increased angiogenic tubule formation in human umbilical vein endothelial cell (HUVEC) cultures compared to controls [74]. Receptors for advanced glycation end-products (RAGEs) signalling via NF-κB may be partly responsible for mediating the increased VEGF expression by RPE cells exposed to Aβ oligomers [75]. Aβ activation of NF-κB signalling has also been reported to induce NLRP3 inflammasome formation, cytokine production [76] and disrupt RPE tight junctions [77]. All of these may facilitate the recruitment of complement factors and immune cells to the subretinal space, which would in turn drive a state of chronic retinal inflammation.

Aβ has also been studied in the context of other genetic risk factors for AD and AMD. For instance, the interplay between Aβ and APOE has long been the subject of investigation, particularly in relation to the APOE4 isoform in AD [78]. APOE isoforms have been proposed to have differential effects on key aspects of Aβ biology, including (i) binding and clearance [79,80], (ii) oligomer stabilisation [81,82] and (iii) *APP* transcription and Aβ secretion [83]. In the majority of studies, the effect on Aβ biology tends to follow the (APOE4 > APOE3 > APOE2) trend in terms of harmful outcomes. Given that the reverse is true for AMD, the individual and combined roles of APOE and Aβ are likely different in the retina compared to the brain. Aβ co-localisation with inactivated C3b, the presence of CFH in amyloid vesicles and the deposition of membrane attack complexes in drusen suggest an interaction with the complement pathway [64,84]. Additionally, Aβ has been shown to activate the classical and alternative complement pathways directly [85,86] through the binding and inhibition of complement factor I [87] and through the upregulation of complement factor B expression in RPE via inflammatory cytokine production by macrophages/microglia [88].

Therapeutic strategies targeting Aβ to treat AMD are currently an active area of research and clinical development. RN6G (PF-04382923), a monoclonal antibody targeting the C-termini of Aβ_1–40_ and Aβ_1–42_ peptides, showed signs of efficacy in mouse models, restoring normal visual function and histological profiles in *TRE4* mice on a high-fat diet [89,90]. RN6G was well-tolerated in a Phase I trial (NCT01003691), but Phase II trials were terminated early due to insufficient participant recruitment. GSK933776 is another Aβ-targeting antibody that binds to the N-terminus of Aβ peptides. However, in a Phase II study of 191 patients, GSK933776 showed no benefit in patients with GA secondary to AMD (NCT01342926) [91]. Finally, GAL-101 (formerly MRZ-99030) is a d-tryptophan and 2-amino-2-methylpropionic acid dipeptide that prevents Aβ oligomer formation by instead forming amorphous, non-amyloidogenic aggregates [92]. GAL-101 eyedrops were well-tolerated in an initial Phase I trial in patients with glaucoma (NCT01714960), and Phase II trials are under development for glaucoma and dry AMD.

Thus far, data is limited on therapies targeting Aβ in AMD, with the exception of the failure of GSK933776. Much of the evidence supporting the role of Aβ in AMD pathogenesis comes from immunohistochemical studies, in which the choice of antibody and a full understanding of its specificity is crucial, and only a static picture for each individual at a single point in time can be achieved. Attempts to address these challenges and glean insights into Aβ dynamics in AMD have come in the form of rodent models, wherein the exogenous delivery of either purified or AAV-delivered Aβ peptides elicit AMD-like pathology [93,94,95]. As rodents lack anatomical maculae, rodent models may not recapitulate all facets of AMD and translate to humans. Furthermore, fundamental differences in retinal aging between mice and primates have also been identified, with variation in Aβ deposition patterns over time [96]. Many Aβ-targeting therapies in AD perform well in rodents, only to fall short in human trials [97]. This highlights the need for greater understanding of the complex nature of the interactions between *APOE* and Aβ in both AD and AMD.

## 5. Link between Lipoprotein Metabolism and Retinal Inflammation

In the current conception of lipid exchange between the RPE and plasma, lipoproteins in the plasma deliver lipophilic essentials to the basolateral surface of the RPE, while the RPE offloads unwanted lipids derived from the phagocytosis of outer segments at the apical surface to the systemic circulation [8]. In AMD, however, this lipid exchange becomes unbalanced due to a combination of factors, including the age-related decline in RPE endo-/exocytotic function and the atherosclerotic impairment of lipid exchange across the Bruch’s membrane–choriocapillaris endothelium (Figure 1a).

Once formed, drusenoid deposits impair outer retinal metabolic exchange and act as foci for retinal inflammation (e.g., via the activation of resident microglia, infiltrating macrophages and complement factors), leading to RPE/photoreceptor degeneration and choroidal neovascularisation (Figure 1b). It has been hypothesised that much of the lipid content of soft drusen is derived from photoreceptor outer segment membranes (rich in esterified cholesterol) phagocytosed by the RPE [8], while other components (e.g., complement factors) likely originate from the plasma, although activated microglia and RPE have also been shown to secrete complement factor H [98,99].

RPD can be seen in around 6% of eyes with early AMD, 26% of intermediate AMD, 36% of geographic atrophy and 19% of neovascular AMD (especially retinal angiomatous proliferation), with a mean age of 79 years, which is 4 years older than the mean age of AMD patients without RPD [16]. Interestingly, RPD are particularly associated with the rs10490924 (c.205G>T, p.Ala69Ser) polymorphism in *ARMS2*, one of the strongest genetic risk factors for AMD (with a relative risk of 8.1 for homozygotes). *ARMS2* is a primate-specific gene. It has been shown to be expressed on the surface of human monocytes and retinal microglia in response to oxidative stress, and in vitro experiments suggest that wildtype ARMS2 protein could opsonise cellular debris for phagocytic clearance via complement C3b [100]. This could explain why loss-of-function AMD-associated SNPs in *ARMS2* may lead to the accumulation of subretinal drusenoid deposits. The physiological role of *ARMS2* appears to contrast with that of *CFH*, a secreted protein which blinds to glycosaminoglycans on cell surfaces to inhibit complement activation [101] and promote the phagocytosis of cellular debris by monocytic phagocytes via CD11b [102].

The effects of drusenoid deposits on retinal physiology are multifaceted. First, drusen could act as barriers to the diffusion of oxygen, nutrients and lipids between the RPE and choriocapillaris, leading to decreased RPE function and survival. Second, drusenoid material (e.g., proteins with oxidative stress-associated carboxyethylpyrrole (CEP) adducts and complement component C1q) may be cytotoxic as drusen isolated from AMD donor retinae have been shown to activate NLRP3 inflammasome in adjacent myeloid cells [103]. Third, and perhaps most importantly, BLinD and SDD drusen components may provide chemoattractant stimuli for plasma-derived macrophages and resident microglia, respectively. For instance, 7-ketocholesterol, a cholesterol photo-oxidation product found in soft drusen, has been shown to have chemotactic effects on microglia, causing them to become activated, migrate to the subretinal space and secret angiogenic factors [104]. Under physiological conditions, microglia reside in a resting state within the inner and outer plexiform layers of the retina, while their presence in the subretinal space is actively inhibited by pro-apoptotic and immune checkpoint signalling from the RPE. For instance, thrombospondin-1 (TSP-1) secreted by the RPE has been shown to induce the homeostatic elimination of microglia via CD47 [98]. In AMD, however, the immune privilege of the subretinal space appears to be perturbed, as IBA1-positive microglia and macrophages have been found in and around large soft drusen and areas of geographic atrophy [45], and around most reticular pseudodrusen [17]. The AMD-associated *CFH*(Y402H) variant has been shown to provide toxic gain-of-function over wildtype *CFH* by increasing the persistence of MPs in the subretinal space through stronger binding to CD11b, which in turn obstructs TSP-1 binding to CD47 within the same lipid rafts [98]. The build-up of subretinal microglia or macrophages could secrete pro-inflammatory cytokines and create a state of chronic low-grade inflammation (or parainflammation), which drives RPE and photoreceptor degeneration. For instance, hyper-reflective foci (HF) in the outer nuclear layer and photoreceptor complex visible on OCT in early and intermediate AMD are believed to represent melanin-containing activated retinal macrophages and have been associated with progression to geographic atrophy and with AMD-risk alleles in *ARSM2*, *CFH* and *APOE* [105,106,107].

Interestingly, APOE2 also appears to feed into this pathogenic pathway by facilitating the survival of monocytic phagocytes in the subretinal space. The previously mentioned *TRE2* mice carrying human *APOE2* demonstrate the accumulation of sub-RPE debris and subretinal mononuclear phagocytes with age [45,108]. The *APOE2*-allele appears to be associated with increased APOE expression but reduced APOE uptake, which leads to the excessive extraction of cholesterol from the lipid rafts of monocytic phagocytes and promotes their activation. In contrast, *APOE4* appeared to be associated with reduced APOE expression and to be less efficient at extracting cholesterol from lipid rafts, which tends to stabilise the myeloid cells [46]. Moreover, the deletion of *Cfh* nearly completely prevented the age- and light stress-induced accumulation of subretinal monocytic phagocytes in the *TRE2* mice [98]. This is not in keeping with Cfh exerting its role in AMD pathogenesis simply via the inhibition of complement activation. Instead, the findings from the mouse models would suggest that Cfh acts downstream of APOE in the regulation of myeloid cell persistence in the subretinal space, which is potentially compatible with the aforementioned role of APOE in recruiting opsonising complement components to drusen.

## 6. Conclusions

Clarifying the interplay between different genetic risk factors and how they contribute to retinal inflammation and drusen formation in AMD is vital for developing targeted, efficacious treatments for this irreversible and blinding disease. Many potential interventions to prevent advanced AMD are currently being investigated. These include the administration of drugs or genetic therapies that modulate retinal complement activation, provide neurotrophic support to photoreceptors [109] and cell therapy to replenish the degenerate RPE/Bruch’s membrane [110]. Recent advances in our understanding of the mechanisms of retinal inflammation contributing to AMD highlight potential new avenues for therapeutic intervention in early and intermediate AMD. These could disrupt drusen formation, target pathogenic monocytic phagocyte activation or enhance monocytic phagocyte clearance from the subretinal space. Deeper understanding of how genetic and environmental risk factors fit into the overall pathogenic mechanism of AMD will continue to guide these developments. In particular, further studies into key components involved in lipid metabolism and myeloid cell activation in the retina will extend this insight.

## Figures and Tables

**Table 1:** Comparison of composition of soft drusen and reticular pseudodrusen.

Composition	Basal Linear Deposits (BLinD) or Soft Drusen	Subretinal Drusenoid Deposit (SDD) or Reticular Pseudodrusen
Lipids	Phospholipid, triglyceride, esterified and unesterified cholesterol	Phospholipid, triglyceride unesterified cholesterol
Apolipoproteins	B, E, A-I, C-I and C-II	E
Complement factors	C5, C5b-9 (membrane attack complex) and CFH	CFH
Other proteins	Vitronectin (low), annexins, crystallins, immunoglobulins and amyloid-β peptides	Vitronectin (high) and opsins
Minerals	Hydroxyapatite	
